# Exogenous H_2_S contributes to recovery of ischemic post-conditioning-induced cardioprotection by decrease of ROS level via down-regulation of NF-κB and JAK2-STAT3 pathways in the aging cardiomyocytes

**DOI:** 10.1186/s13578-016-0090-x

**Published:** 2016-04-18

**Authors:** Lina Li, Meixiu Li, Youyou Li, Weiming Sun, Yuehong Wang, Shuzhi Bai, Hongxia Li, Bo Wu, Guangdong Yang, Rui Wang, Lingyun Wu, Hongzhu Li, Changing Xu

**Affiliations:** Department of Pathophysiology, Harbin Medical University, Baojian Road, Harbin, 150081 China; The Key Laboratory of Cardiovascular Medicine Research, Harbin Medical University, Ministry of Education, Harbin, China; The Cardiovascular and Metabolic Research Unit, Laurentian University, Sudbury, ON Canada

**Keywords:** Hydrogen sulfide, Post-conditioning, Oxidative stress, Aging cardiomyocytes

## Abstract

**Background:**

Hydrogen sulfide (H_2_S), a third member of gasotransmitter family along with nitric oxide and carbon monoxide, generated from mainly catalyzed by cystathionine-lyase, possesses important functions in the cardiovascular system. Ischemic post-conditioning (PC) strongly protects against the hypoxia/reoxygenation (H/R)-induced injury and apoptosis of cardiomyocytes. However, PC protection is ineffective in the aging cardiomyocytes. Whether H_2_S restores PC-induced cardioprotection by decrease of reactive oxygen species (ROS) level in the aging cardiomyocytes is unknown.

**Methods:**

The aging cardiomyocytes were induced by treatment of primary cultures of neonatal cardiomyocytes using d-galactose and were exposed to H/R and PC protocols. Cell viability was observed by CCK-8 kit. Apoptosis was detected by Hoechst 33342 staining and flow cytometry. ROS level was analyzed using spectrofluorimeter. Related protein expressions were detected through Western blot.

**Results:**

Treatment of NaHS (a H_2_S donor) protected against H/R-induced apoptosis, cell damage, the expression of cleaved caspase-3 and cleaved caspase-9, the release of cytochrome *c* (Cyt *c*). The supplementation of NaHS also decreased the activity of LDH and CK, MDA contents, ROS levels and the phosphorylation of IκBα, NF-κB, JNK2 and STAT3, and increased cell viability, the expression of Bcl-2, the activity of SOD, CAT and GSH-PX. PC alone did not provide cardioprotection in H/R-treated aging cardiomyocytes, which was significantly restored by the addition of NaHS. The beneficial role of NaHS was similar to the supply of N-acetyl-cysteine (NAC, an inhibitor of ROS), Ammonium pyrrolidinedithiocarbamate (PDTC, an inhibitor of NF-κB) and AG 490 (an inhibitor of JNK2), respectively, during PC.

**Conclusion:**

Our results suggest that exogenous H_2_S contributes to recovery of PC-induced cardioprotection by decrease of ROS level via down-regulation of NF-κB and JAK2/STAT3 pathways in the aging cardiomyocytes.

## Background

Hydrogen sulfide (H_2_S) has been recognized as a physiological mediator with a variety of functions [1]. Endogenous H_2_S production is mainly catalysed by cystathionine β-synthase (CBS), cystathionine-γ-lyase (CSE) and 3-mercaptosulphurtransferase (3-MPST) [2]. Among them, CSE is a key enzyme in the production of endogenous H_2_S in the cardiovascular system and the deficiency of CSE leads to decreased endogenous H_2_S level and appeared a series of diseases in the cardiovascular system [3–5]. As an important gasotransmitter in the cardiovascular system, H_2_S plays an important role of inhibiting cardiovascular diseases, such as anti-ischemia/reperfusion (I/R), anti-atherosclerosis, anti-inflammation, vasodilatation, anti-oxidation, etc. by regulating protein modification, signaling pathways, ion channel protein and metabolism [2–6].

Myocardial ischemia (hypoxia)/reperfusion (reoxygenation) causes cardiomyocytes injury, including cardiomyocytes apoptosis and necrosis by increase of free radical generation, calcium overload and the adhesion of leukocytes [7, 8]. Reactive oxygen species (ROS) are increased during I/R then lead to cell death [9]. The signaling pathways involved in ROS generation, such as nuclear actor–kappa B (NF-κB) and janus kinase-2 (JAK2)-signal transducer and activator of transcription 3 (STAT3) pathways, have been investigated intensively [10, 11]. As we all know, ischemic post-conditioning (PC) is one of the most powerful endogenous cardioprotective mechanisms [12, 13]. PC can inhibit I/R-induced cardiomyocytes injury via decrease of ROS. However, the beneficial effect of PC has been documented in the young heart of every species tested [13, 14], but this response is attenuated or loss in the aged heart [8, 9, 15, 16].

It was previously reported that H_2_S is involved in PC-induced myocardial protective effects in the young hearts [17]. Our previous study indicated that exogenous H_2_S recovered cardioprotection from PC by activation of PI3K-Akt-GSK-3β pathway in isolated aging rat hearts and via inhibiting mPTP opening in the aging cardiomyocytes [8, 9]. However, whether H_2_S plays a key role in the recovery of PC-induced cardioprotection by decrease of ROS level via down-regulating NF-κB and JAK2-STAT3 pathways in the aging cardiomyocytes is unknown. In the present study, we first evaluated the effect of PC on ROS-induced injury and apoptosis during hypoxia/reoxygenation (H/R) in the aging cardiomyocytes. Next, we examined whether exogenous H_2_S restored PC protection though inhibiting ROS generation in the aging cardiomyocytes. Furthermore, we focused on the mechanism of ROS generation, including NF-κB and JAK2-STAT3 pathways, and the role that exogenous H_2_S played during this process.

## Methods

### Materials

Sodium hydrogen sulfide (NaHS), N-acetyl-cysteine (NAC, an inhibitor of reactive oxygen species, ROS), Ammonium pyrrolidinedithiocarbamate (PDTC, a NF-κB inhibitor), AG 490 (a JNK2 inhibitor) were purchased from Sigma Chemical Co. (St. Louis, MO, USA). The anti-cleaved caspase-3 and -9, Bcl-2, cytochrome c (Cyt *c*), and GAPDH were from proteintech (Wuhan, China). Hoechst 33342 were from Santa Cruz (Bergheimer, Germany). The anti-IκBα-NF-κB and JNK2-STAT3 antibodies were obtained from Cell Signaling Technology (Danvers, USA). Assay kits for malondialdehyde (MDA), superoxide dismutase (SOD), Glutathione peroxidase (GSH-PX), catalase (CAT), lactate dehydrogenase (LDH), creatine kinase (CK) and ROS were purchased from Nanjing Jiancheng Bioengineering Institute (Nanjing, China). All other chemicals were from Sigma or Santa Cruz.

### Primary culture of cardiomyocytes

Primary cultures of neonatal cardiomyocytes were prepared as previously described [7, 8]. Newborn Wistar rats, aged 1–3 days and weighing 5–8 g, were used for this study. All animal experiments were conducted in compliance with the Guide for the Care and Use of Laboratory Animals published by the China National Institutes of Health and approved by the Animal Care Committees of Harbin Medical University, China. Briefly, cells were dissociated from minced hearts of 1- to 3-day neonatal Wistar rats with a 0.25 % solution of crude trypsin. Cells were cultured as monolayers at a density of 5 × 10^4^ cells/cm^2^ in Dulbecco’s modified Eagle medium (DMEM) equilibrated with humidified air containing 5 % CO_2_ at 37 °C. The medium contained 10 % calf serum and 2 μM fluorodeoxyuridine, the latter to prevent proliferation of non-myocytes.

### The aging cardiomyocytes induced by d-galactose and established model of hypoxia/reoxygenation

The treatment for d-galactose induction was as previously described [8, 18, 19]. Briefly, 10 g/L d-galactose was added to the cardiomyocytes in the culture cluster for 48 h.

A hypoxic condition was produced by D-Hank solution saturated with 95 % N_2_ and 5 % CO_2_. The pH was regulated to 6.8 with lactate to mimic ischemic solution. The aging cardiomyocytes were put into a hypoxic incubator that was equilibrated with 1 % O_2_/5 % CO_2_/94 % N_2_. After hypoxia, the culture medium was rapidly replaced with fresh DMEM with 10 % fetal bovine serum (normoxic culture solution) for initiating reoxygenation.

### Experimental protocols

**Fig. 1:**
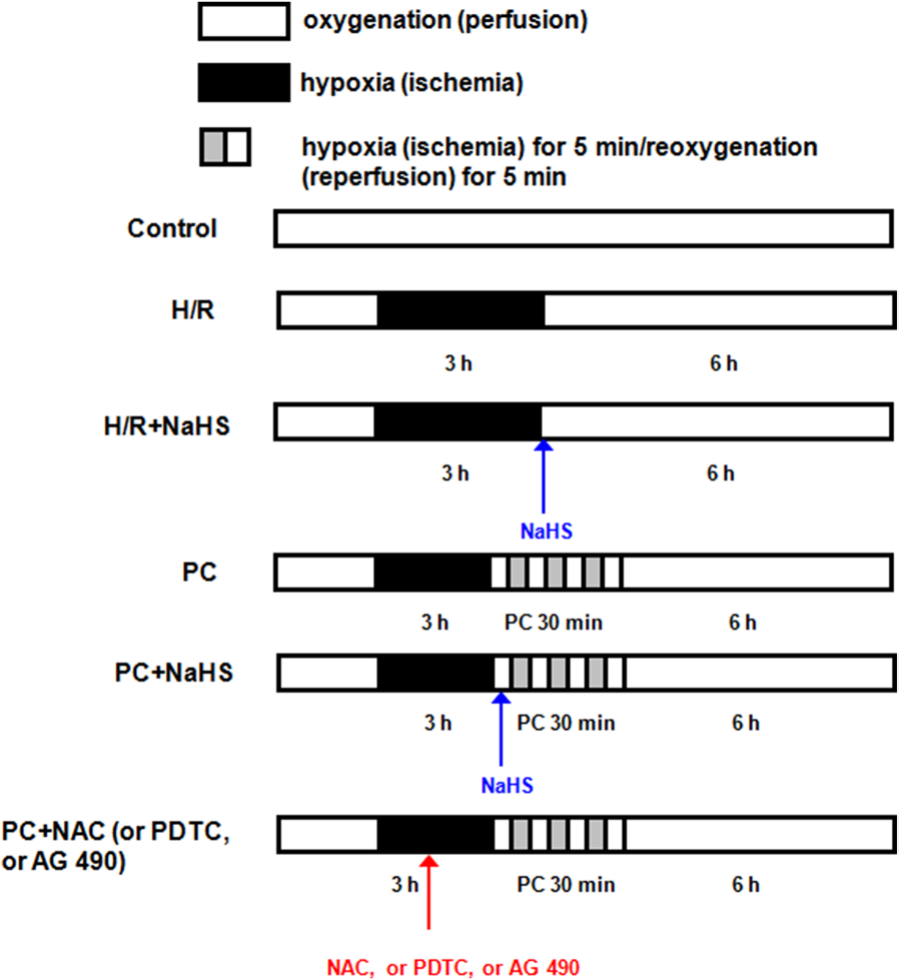
Summary of experimental treatments protocol. The aging cardiomyocytes were exposed to hypoxic culture medium for 3 h and reoxygenated for 6 h by replacing the hypoxic culture medium with fresh DMEM with 10 % fetal bovine serum. For details of ischemic post-conditioning, NaHS, NAC, PDTC and AG 490 treatments see text

The aging cardiomyocytes were randomly divided into the following six groups. Each group included eight samples (n = 8) (Fig. 1): (1) Control group (Control): The aging cardiomyocytes were cultured for 9 h with 10 % fetal bovine serum-DMEM; (2) Hypoxia/reoxygenation group (H/R): The aging cardiomyocytes were exposed to hypoxic culture medium for 3 h and reoxygenated for 6 h by replacing the hypoxic culture medium with fresh DMEM with 10 % fetal bovine serum; (3) H/R + NaHS group: The procedure was similar to that for group 2, except that 100 μM NaHS were added in 6 h reoxygenation; (4) PC group: At the end of 3 h of hypoxia, the aging cardiomyocytes were exposed to normoxic culture solution for 5 min, after which cells were placed in hypoxic solution for 5 min. The PC cycle was repeated three times and followed by 6 h of reoxygenation; (5) PC + NaHS group: At the end of 3 h of hypoxia, initiated immediately at the onset of reoxygenation, 100 μM NaHS were given at the onset of reoxygenation for 5 min following with 5 min hypoxia. This protocol was repeated for another two times. The cells were then treated as those of group 3; (6) PC + NAC (or PDTC, or AG490) group: 5 mM NAC (or 100 μM PDTC or 100 μM AG490) were added to the medium 40 min before the end of hypoxia. The cells were then treated as those of group 4.

### Cell viability assay

Cell viability was measured by Cell Counting Kit-8 (CCK-8). Cells were seeded in 96-well plates at a concentration of 3 × 10^3^ cells/well. After 24 h of each treatment, 10 μl was added to each well of CCK-8 immediately. Subsequently, they were incubated for 2 h at 37 °C. Using a microplate spectrophotometer, the plates were read at 570 nm (A570) to determine their optical density.

### Measurement of MDA level, LDH, CK, CAT,GSH-PX and SOD activities

The activity of LDH, CK, SOD, CAT and GSH-PX and the content of MDA in the cell culture medium were measured spectrophotometrically with a commercially available assay kit according to manufacturer’s introduction.

### Apoptotic aate of cells by flow cytometry assay and Hoechst 33342 staining

The apoptotic rate was measured by flow cytometry as described previously [7, 8]. Cells were washed three times with ice-cold PBS, and then stained with annexin V-fluorescein isothiocyanate for 15 min at room temperature in 200 μl binding buffer. Next, 300 μl binding buffer was added, and the cells were stained with propidium iodide for 30 min at 4 °C. The fluorescence of the cells was analyzed by flow cytometry. The percentage of apoptotic cells was determined using Mod Fit LT software (Verity Software House Inc., Topsham, ME, USA).

Cells were analyzed for apoptosis after visualization of nuclei morphology with fluorescent DNA-binding dye Hoechst 33342, as described previously [8]. After treatment, cells were rinsed with PBS and incubated with 5 μg/ml Hoechst 33342 for 10 min. Nuclei were visualized at 400× magnification using fluorescent microscopy at an excitation wavelength of 330–380 nm. Apoptotic nuclei of cells were assessed by counting the number of cells that displayed nuclear morphology changes, such as chromatin condensation and fragmentation.

### Measurement of intracellular ROS level

ROS generation was estimated using a ROS assay kit. Cells were seeded in 12-well plate (1 × 10^6^ cells/sample) and exposed to different treatments. Intracellular ROS oxidizes non-fluorescent DCFH into fluorescent DCF, and then observed by a fluorescence microscope. The ROS level was reflected by the fluorescence intensity.

### Western blotting analysis

The related protein expressions was measured by Western blot as described previously [7, 8, 13]. Brifely, equal amounts of proteins were subjected to sodium dodecyl sulfatepolyacrylamide gel electrophoresis and blotted on polyvinylidene fluoride membranes. The membranes were incubated with antibodies against cleaved-3 and -9, Bcl-2, p-IκBα/t- IκBα, p-NF-κB/t-NF-κB and GAPDH. The secondary antibody was goat anti-rat immunoglobulin G. The intensities of the protein bands were quantified by a Bio-Rad ChemiDoc™ EQ densitometer and Bio-Rad Quantity One software (Bio-Rad Laboratories). The protein concentration was quantified using the BCA Protein Assay kit (Beyotime, Nantong, China).

### Detection of Cyt *c* release from mitochondrial

Western blot analysis of Cyt *c* in the cytosolic fraction was performed as described previously [7, 8]. Briefly, cells were harvested, washed twice with ice-cold PBS, and incubated in ice-cold Tris-sucrose buffer (0.35 mM sucrose, 10 mM Tris–HCl at pH 7.5, 1 mM EDTA, 0.5 mM dithiothreitol, 0.1 mM phenylmethylsulphonyl fluoride). After a 40 min incubation, cells were centrifuged at 1000×*g* for 5 min at 4 °C and the supernatant was further centrifuged at 40,000×*g* for 30 min at 4 °C. The supernatant was retained as the cytosolic fraction and analyzed by Western blot with a primary rat anti-Cyt *c* monoclonal antibody and a secondary goat anti-rat immunoglobulin G (Promage). GAPDH expression was used as the control.

### Statistical analysis

All data were expressed as the mean ± SE and represented at least three independent experiments. Statistical comparisons were made using student’s t test or one-way ANOVA followed by a post hoc analysis (Tukey test) where applicable. Significance level was set at p < 0.05.

## Results

### The change of cell viability and LDH and CK activities

**Fig. 2:**
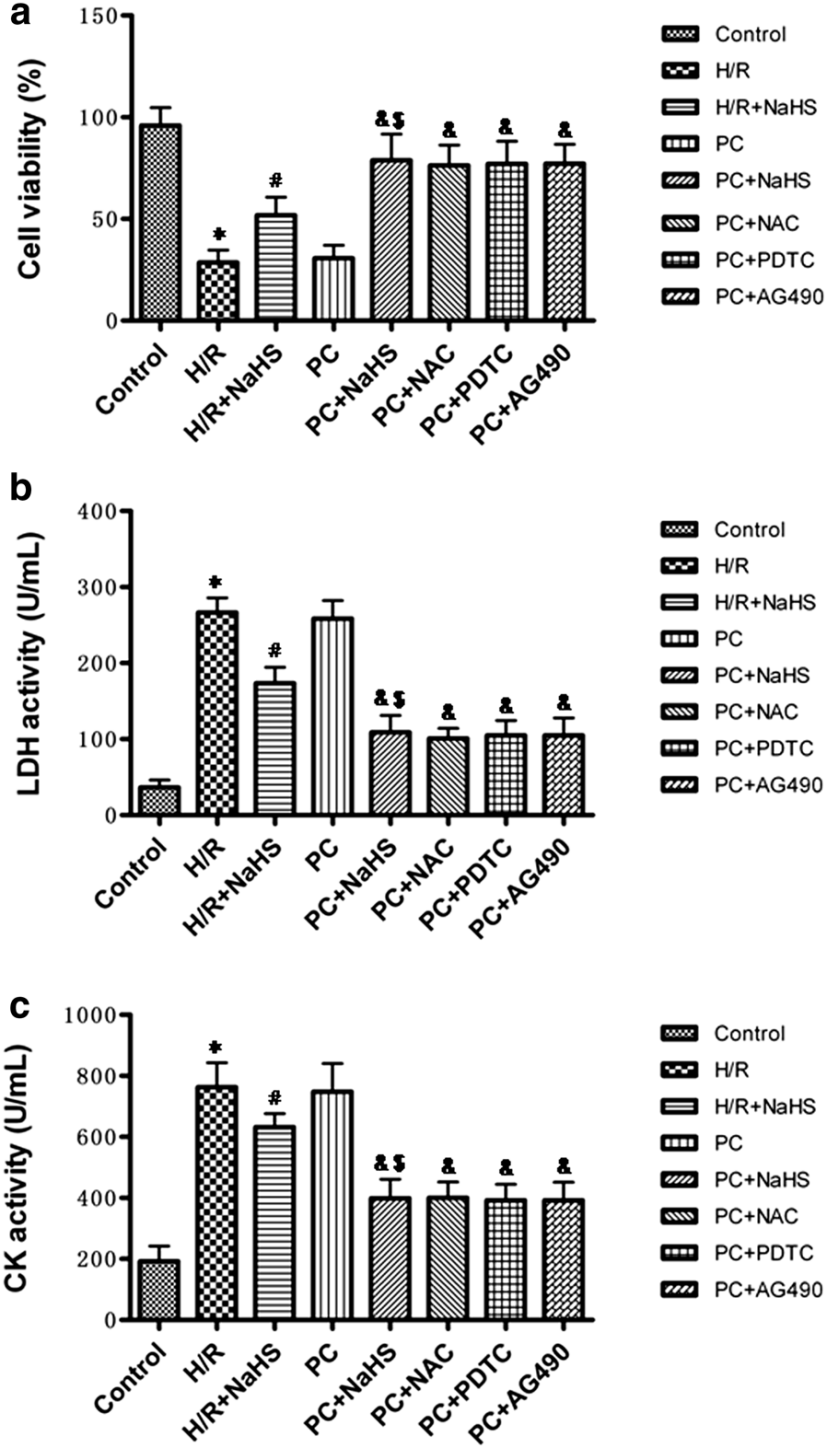
The effect of exogenous H_2_S on the cell viability and LDH and CK activities. **a** Cell viability was measured by CCK-8 kit. LDH (**b**) and CK (**c**) activities were detected in the cell culture medium. All data are mean ± SEM of eight determinations. *p < 0.05 vs. control group; ^#^p < 0.05 vs. H/R group; ^&^p < 0.05 vs. PC group; ^$^p < 0.05 vs. H/R + NaHS group

Cell viability was reduced and the activity of LDH and CK was increased in the H/R group (p < 0.05 versus control group) and could be reversed by NaHS co-treatment. The cell viability of the PC group was similar to the H/R group. Compared with the H/R + NaHS group, cell viability was obviously increased in the PC + NaHS group (p < 0.05), The beneficial role of PC + NaHS on the these indexes was similar to PC + NAC (or PDTC, or AG 490), respectively (Fig. 2a–c).

### The change of apoptosis

**Fig. 3:**
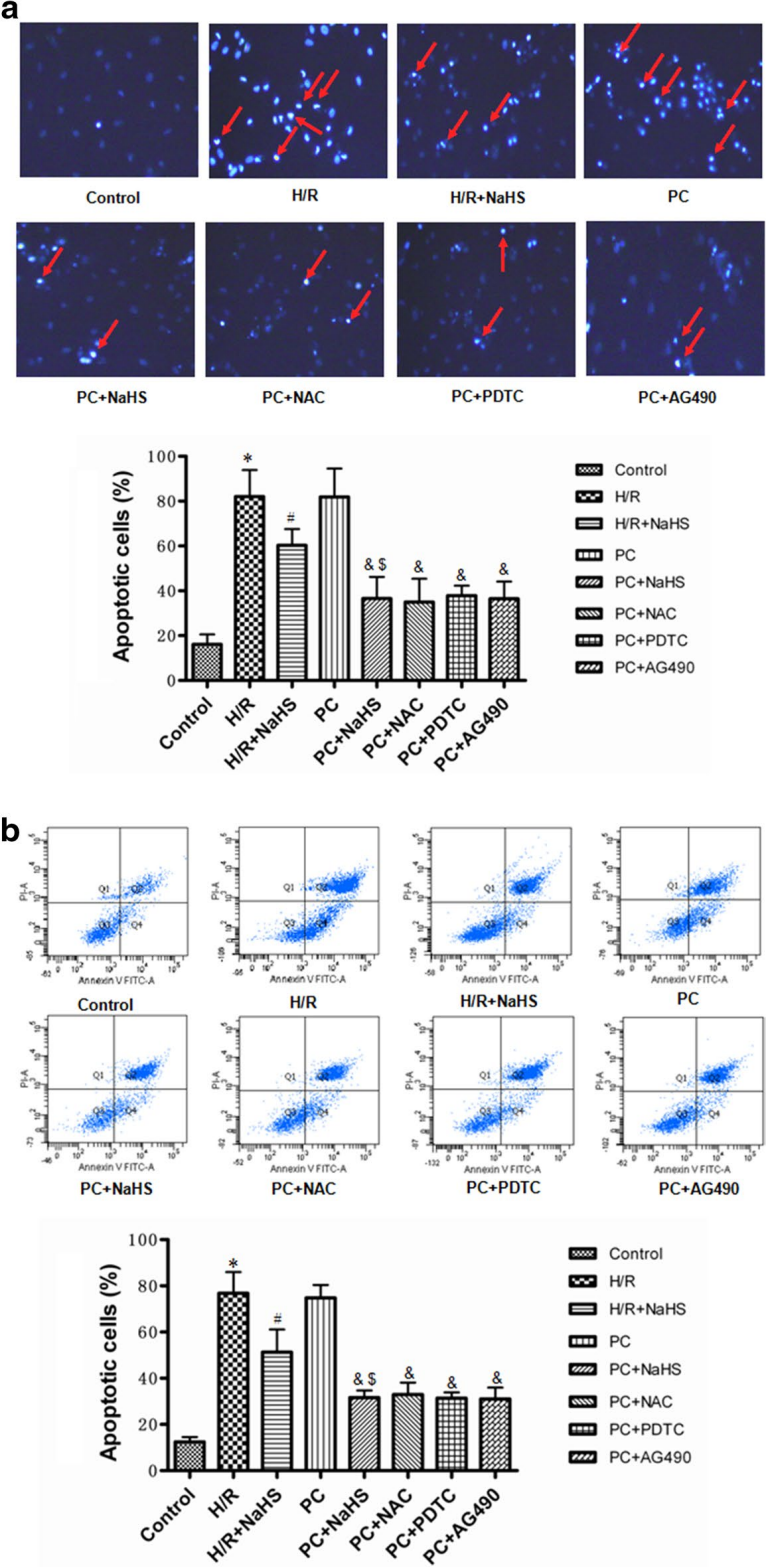
The effect of exogenous H_2_S on the apoptosis. **a** Detection of nuclear morphology in apoptotic cells by Hoechst 33342 staining. Apoptotic cells were identified as cells with condensed, disrupted nuclei (arrow, Hoechst staining, ×400). *Scale bar* = 100 μm. Apoptotic cells in at least five random fields were counted. **b** Apoptosis analyzed by flow cytometry. All data were from four independent experiments. *p < 0.05 vs. control group; ^#^p < 0.05 vs. H/R group; ^&^p < 0.05 vs. PC group; ^$^p < 0.05 vs. H/R + NaHS group

Results of flow cytometry assay and Hoechst 33342 staining showed that H/R significantly increased the apoptosis rate than that in control group (p < 0.05). H/R + NaHS makedly decreased the apoptosis rate (p < 0.05 versus the H/R group). The apoptosis rate of the PC group was similar to the H/R group. Compared with the H/R + NaHS group, the apoptosis rate was further decreased in the PC + NaHS group. The effect of PC + NaHS on the apoptosis rate was similar to PC + NAC (or PDTC, or AG 490), respectively (Fig. 3a–b).

### The change of apoptotic relative factors

**Fig. 4:**
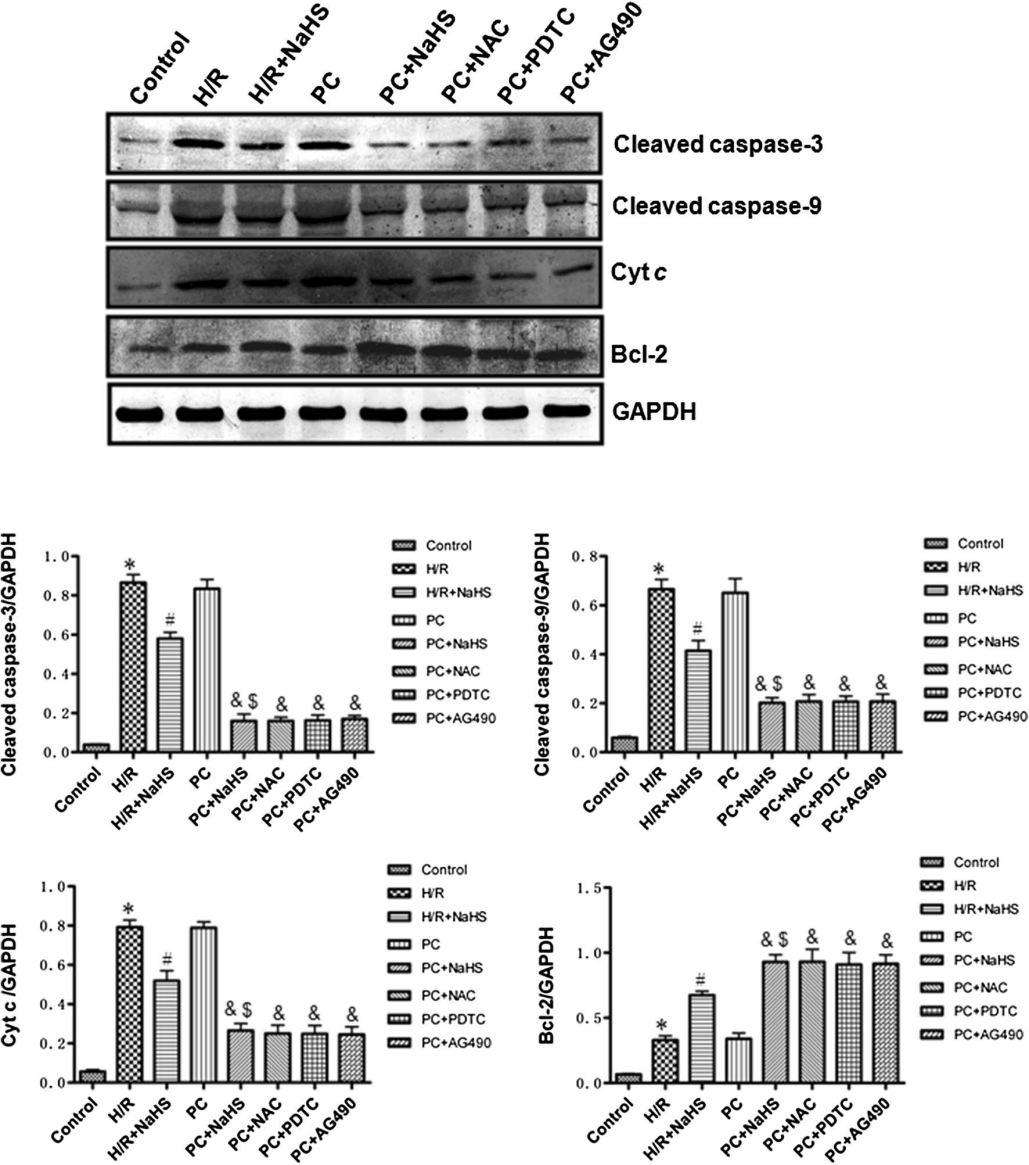
The effect of exogenous H_2_S on the expression of Bcl-2, cleaved caspase-3 and cleaved caspase-9, cytosolic Cyt *c*. The intensity of each band was quantified by densitometry, and data were normalized to the GAPDH signal. All data were from four independent experiments. *p < 0.05 vs. control group; ^#^p < 0.05 vs. H/R group; ^&^p < 0.05 vs. PC group; ^$^p < 0.05 vs. H/R + NaHS group

Figure 4 showed that the expression of pro-apoptotic factors (cleaved caspase-3, cleaved caspase-9 and Cyt *c*) and anti-apoptotic factors (Bcl-2) was increased in the H/R group compared with the control group (p < 0.05). Compared with H/R, H/R + NaHS decreased expression of pro-apoptotic factors but increased expression of anti-apoptotic factors (p < 0.05). The results of the PC group were similar to those of the H/R group. PC + NaHS treatment significantly decreased the expression of pro-apoptotic factors and increased the expression of anti-apoptotic factors in comparison with the H/R + NaHS (p < 0.05). The effect of PC + NaHS on apoptotic relative factors was similar to PC + NAC (or PDTC, or AG 490), respectively (Fig. 4).

### The change of oxidative stress related factors

**Fig. 5:**
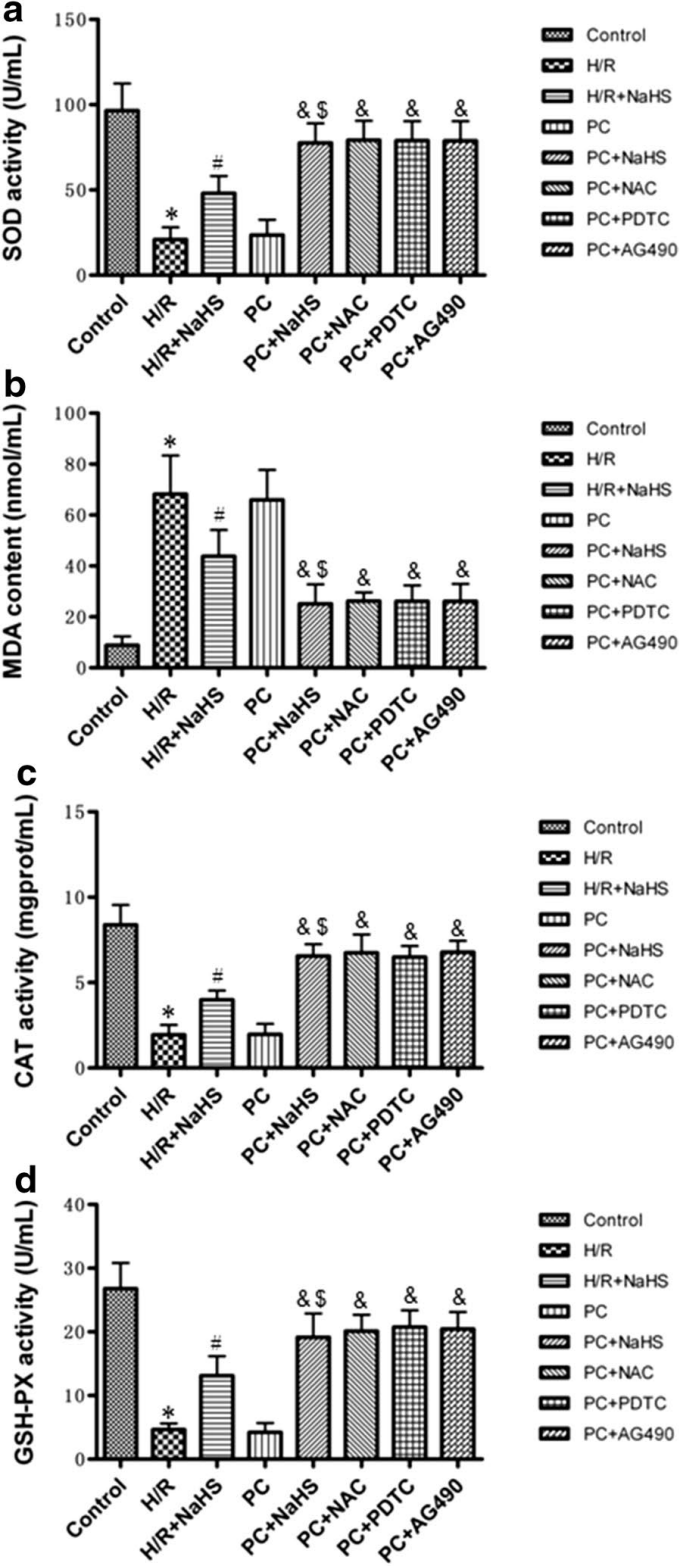
The effect of exogenous H_2_S on MDA content and SOD, CAT and GSH-PX activities. SOD (**a**), CAT (**c**) and GSH-PX (**d**) activities and MDA (**b**) content were detected in the cell culture medium. All data are mean ± SEM of eight determinations. *p < 0.05 vs. control group; ^#^p < 0.05 vs. H/R group; ^&^p < 0.05 vs. PC group; ^$^p < 0.05 vs. H/R + NaHS group

**Fig. 6:**
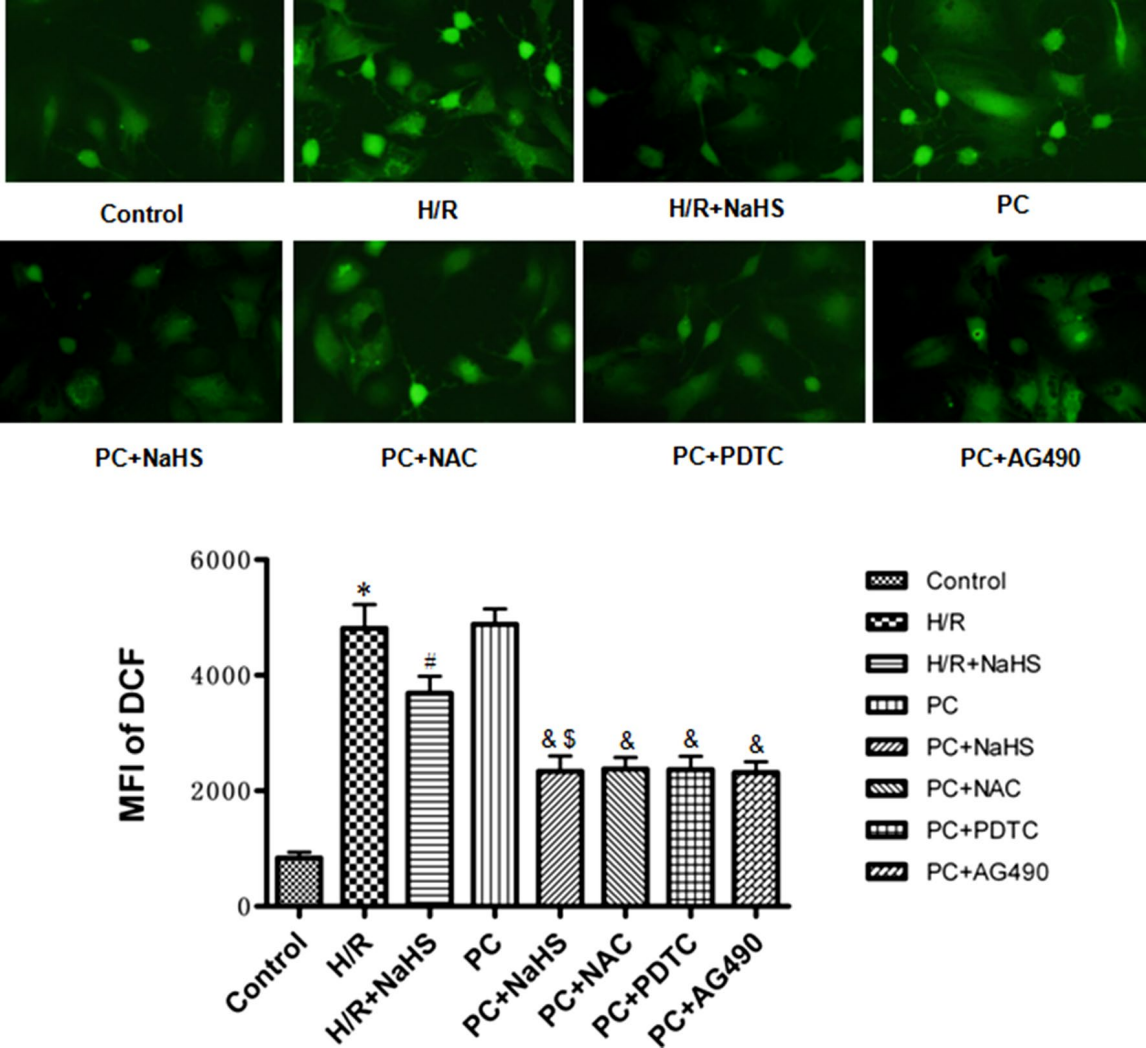
The effect of exogenous H_2_S on ROS levels. DCFH-DA staining followed by photofluorography was carried out to observe intracellular ROS levels. Quantitative analysis of the mean fluorescence intensity (MFI) of DCFH-DA with Image-Pro Plus software. Mean ± SEM, n = 4. *p < 0.05 vs. control group; ^#^p < 0.05 vs. H/R group; ^&^p < 0.05 vs. PC group; ^$^p < 0.05 vs. H/R + NaHS group

MDA contents and ROS levels were increased and the activity of SOD, CAT and GSH-PX was decreased in the H/R group (p < 0.05 versus the control group). MDA contents and ROS levels were decreased and the activity of SOD, CAT and GSH-PX was increased in the H/R + NaHS group (p < 0.05 versus the H/R group). The change of these indexes in the PC group was similar to that in the H/R group. Compared with the H/R + NaHS, PC + NaHS futher decreased the content of MDA and the level of ROS, and increased SOD, CAT and GSH-PX activities (p < 0.05). The effect of PC + NaHS on oxidative stress related factors was similar to PC + NAC (or PDTC, or AG 490), respectively (Figs. 5, 6).

### The change of NF-κB pathway

**Fig. 7:**
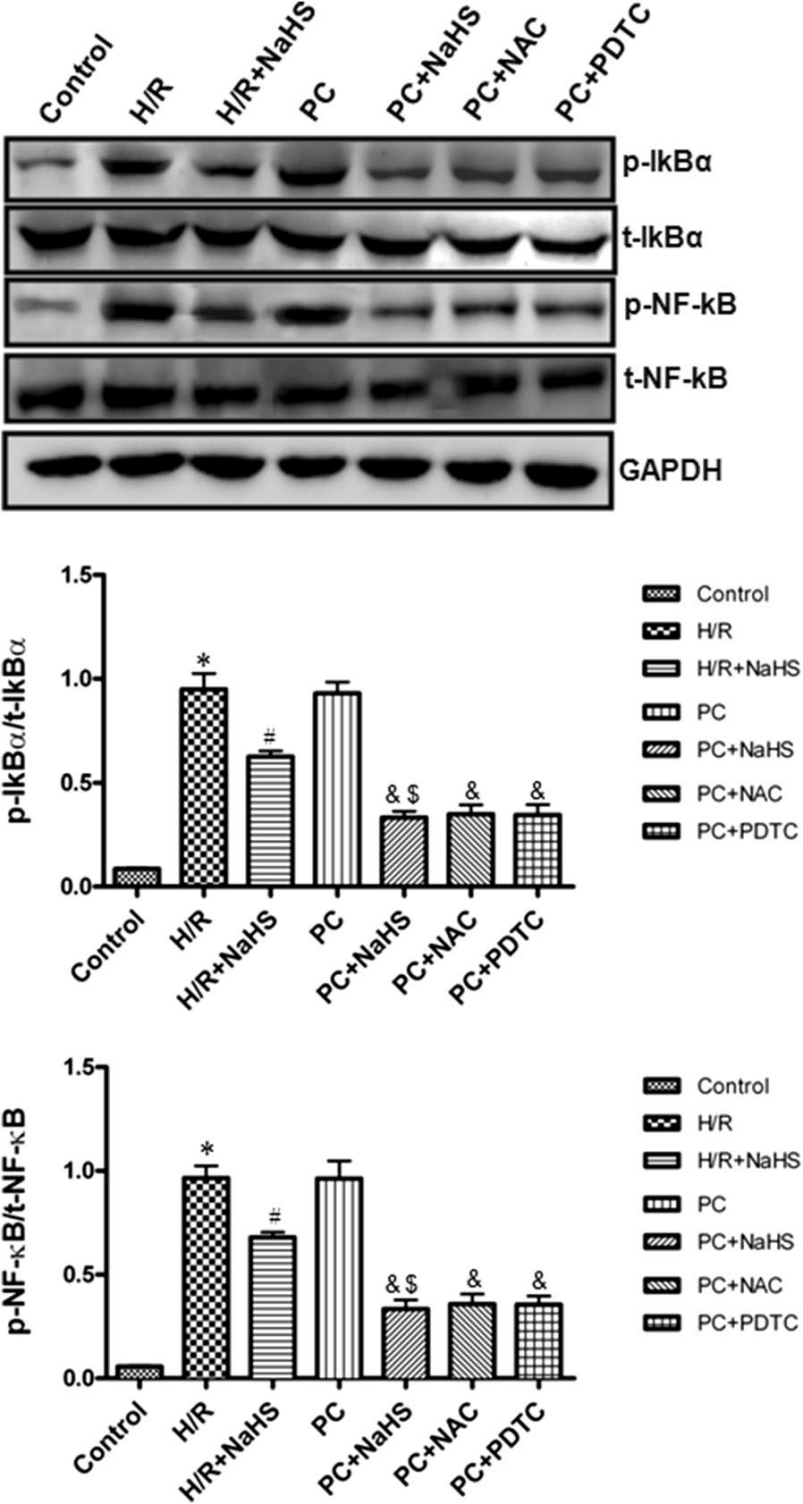
The effect of exogenous H_2_S on the NF-κB pathway. The phosphorylation of IκBα and NF-κB was detected using Western bloting. The graphs represent the optical density of the bands of phosphorylated IκBα and NF-κB normalized with the expression of total IκBα and NF-κB, respectively. All data were from three independent experiments. *p < 0.05 vs. control group; ^#^p < 0.05 vs. H/R group; ^&^p < 0.05 vs. PC group; ^$^p < 0.05 vs. H/R + NaHS group

The activity of phosphorylated IκBα and NF-κB in the H/R group was significantly higher than that in the control group (p < 0.05). The activity of phosphorylated IκBα and NF-κB was markedly lower in the H/R + NaHS group than in the H/R group (p < 0.05). The change of phosphorylated IκBα and NF-κB activities in the PC group were similar to those in the H/R group. In the PC + NaHS group, the activity of phosphorylated IκBα and NF-κB was decreased to a larger extent than that in the H/R + NaHS (p < 0.05). The effect of PC + NaHS on NF-κB pathway was similar to PC + NAC or PC + PDTC, respectively. The total amount of IκBα and NF-κB protein remained unchanged with the different stimulations (Fig. 7).

### The change of JAK2-STAT3 pathway

**Fig. 8:**
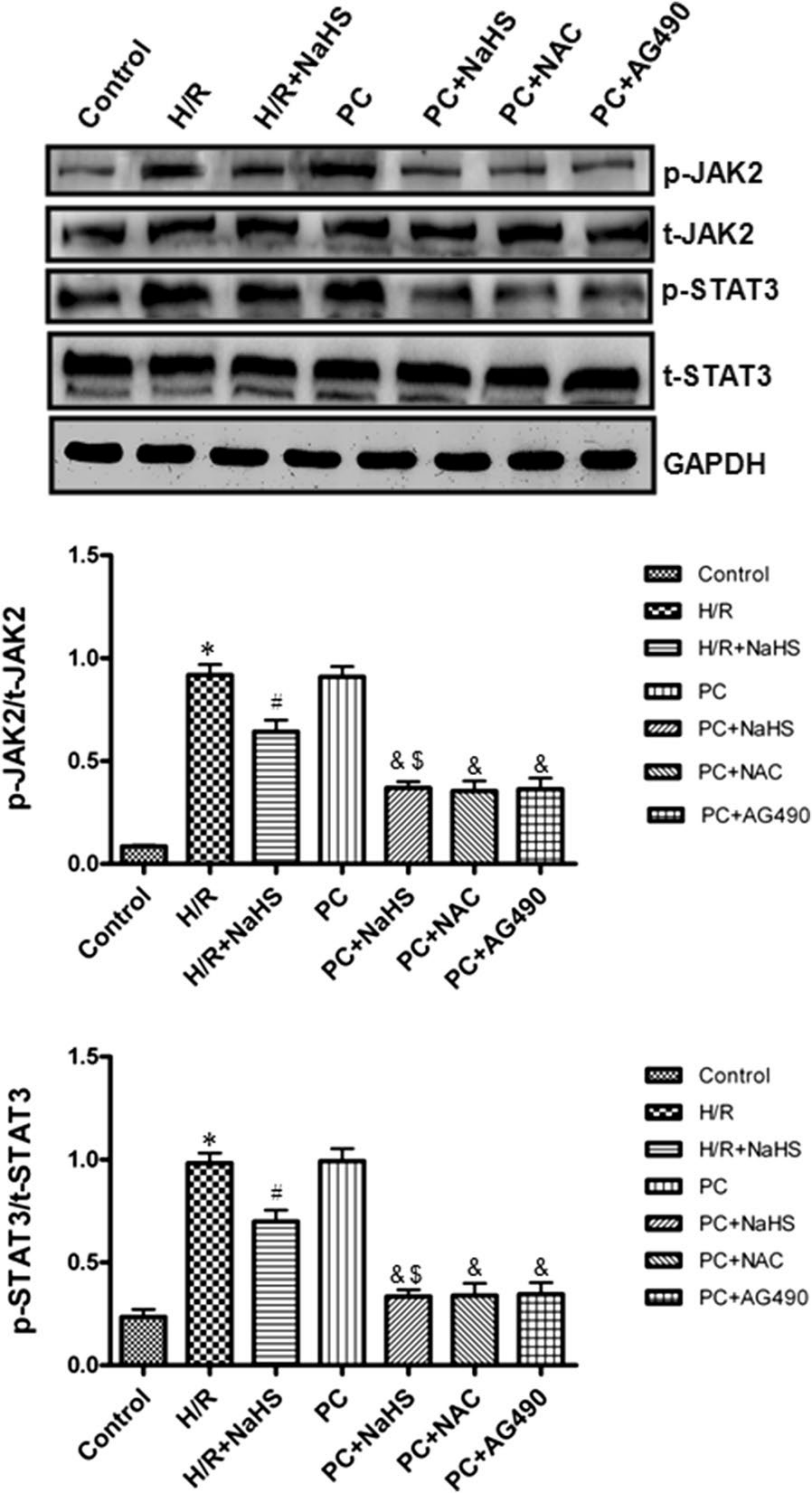
The effect of exogenous H_2_S on the JAK2-STAT3 pathway. The phosphorylation of JAK2 and STAT3 was detected using Western bloting. The graphs represent the optical density of the bands of phosphorylated JAK2 and STAT3 normalized with the expression of total JAK2 and STAT3, respectively. All data were from three independent experiments. *p < 0.05 vs. control group; ^#^p < 0.05 vs. H/R group; ^&^p < 0.05 vs. PC group; ^$^p < 0.05 vs. H/R + NaHS group

Our results showed that the level of phosphorylated JAK2 and STAT3 was up-regulated in the H/R group compared with those in the control group (p < 0.05). The phosphorylation of JAK2 and STAT3 was down-regulated in the H/R + NaHS group compared with that in the H/R group (p < 0.05). Their changes in the PC group were similar to the H/R group. PC + NaHS significantly decreased the phosphorylation of JAK2 and STAT3 in comparison with the H/R + NaHS (p < 0.05). The effect of PC + NaHS on JAK2-STAT3 pathway was similar to PC + NAC or PC + AG 490, respectively. The total amount of JAK2 and STAT3 protein remained unchanged with different stimulations (Fig. 8).

## Discussion

Previously, we detected senescence-associated β-gal (SA β-gal) activity, rat advanced glycation end products (AGEs) content, cell division index and cell cycle proteins (cyclin D1 and p21^Cip/WAF−1^) in treated cardiomyocytes with different concentrations of d-galactose for different times and found that 10 g/L d-galactose for 48 h successfully established the d-galactose-induced aging model of cardiomyocytes [8]. According to reports and our previous results, in the present study, we established cardiomyocytes aging model using 10 g/L d-galactose for 48 h in primary cultures of neonatal cardiomyocytes.

Myocardial H/R causes cardiomyocytes necrosis and apoptosis. The mitochondrial pathway is an important apoptotic pathway [8, 9, 20, 21]. Cyt *c* is the initiating factor of mitochondrial apoptosis pathway. The Cyt *c* is released from injured mitochondria and triggers cytosolic caspase-3 activation through formation of the cytochrome c/Apaf-1/caspase-9-containing complex apoptosome and then lead to apoptosis [8, 9, 20, 21]. Bcl-2 belong to a potent inhibitor of apoptosis and inhibit the mitochondria disruption and the subsequent Cyt *c* release, and the activation of caspase [8, 9, 20, 21]. PC can inhibit H/R-induced cardiomyocytes apoptosis by decrease of the mitochondrial apoptotic pathway [8, 9, 20, 21]. PC protection is mediated by numerous factors, including adenosine, bradykinin, and opioid receptors. These mediators activate downstream kinases, such as ERK1/2, PKC, PI3K/Akt, and p38 MAPK; these mediators all converge at the mitochondria, which act as an integration point that is critical for cardiomyocytes survival [7, 8, 13, 14]. However, it was recently reported that PC loses its myocardial protective effect in ageing hearts [8, 9, 16, 22–24]. The main reason is that aging affects cardiomyocytes at several subcellular and molecular levels, including alterations at the level of the DNA, increased oxidative stress, changes in gene/protein expression and posttranslational modifications, and the handling of cellular ‘waste’ material by autophagy [8, 9, 16, 22–24]. All these alterations decrease the tolerance of cardiomyocytes to stress [8, 9, 16, 22–24]. Our results showed that the difference in cell viability, the apoptotic rate, LDH and CK activities and cleaved caspase-9, cleaved caspase-3, Bcl-2 and Cyt *c* expression between the H/R and PC groups in aging cardiomyocytes was not significant (Figs. 2, 3, 4). These results indicate that PC loses protective role against H/R injury in the in aging cardiomyocytes. It is consistent with our previous results [8, 9, 16].

H_2_S is a kind of new gas molecules and has a lot of physiological function in different tissues and organs [2, 3, 25, 26]. Especially, it plays an important role in the cardiovascular system [2, 3, 25, 26]. Endogenous H_2_S is produced enzymatically via the cysteine metabolic enzymes CSE in the cardiovascular system [2, 3, 25, 26]. Recent studies have shown that increased endogenous CSE/H_2_S pathway expression or exogenous H_2_S is involved in PC-induced cardioprotection in young rat hearts [17]. Our previous data showed that in the aging hearts and cardiomyocytes, the loss of PC-induced cardioprotection is associated with down-regulating the CSE/H_2_S pathway and exogenous H_2_S restored the beneficial effect of PC [8, 9, 16]. The present data showed that PC + NaHS (a H_2_S donor) further enhanced the cardioprotective roles of H/R + NaHS. These further suggest that exogenous H_2_S involves in the recovery of PC-induced cardioprotection in the aging cardiomyocytes (Figs. 2, 3, 4, 5, 6, 7, 8).

H/R induces ROS generation. ROS promotes production of end product of lipid peroxidation, such as MDA [9, 27]. MDA can damage tissues and cells. At the same time, the level of MDA reflects the extent of ROS. The overproduction of ROS can be detoxified by endogenous antioxidants, causing their cellular stores to be depleted [9, 28]. SOD, CAT and GSH-PX, as free radical scavengers, mainly scavenge hydrogen peroxide (H_2_O_2_) and hydroxyl free radicals (OH•), finally reduce cell injury from oxidative stress [9, 29]. A number of signaling pathways involve in generation of ROS. Among them, NF-κB and JAK2-STAT3 pathways are important [10, 11]. NF-κB regulates expression of a variety of genes involved in immune responses, inflammation, proliferation, and programmed cell death (apoptosis) [30]. In homeostatic cells, NF-κB remains in the cytoplasm in its inactive form, associated with proteins that inhibit the κB site called κB inhibitors (IκB). IκBα is one of seven isoforms of IκB [10]. IκBα phosphorylation and degradation promote NF-κB phosphorylation and then increases NF-κB nuclear translocation and transcriptional activity. Translocated NF-κB induces generation of ROS [30]. The JAK2-STAT3 signaling pathway affects cell proliferation, migration, growth, differentiation and death [11, 31]. Meanwhile, the JAK2-STAT3 pathway also is a highly evolutionarily conserved pathway that is involved in growth and development and controls communication among cells, signaling transduction in the cytoplasm and gene transcription in the nucleus [11, 32]. It was recently reported that the JAK2-STAT3 pathway up-regulates oxidative stress and increases ROS levels [11, 33, 34]. Many researches indicated that H/R promoted NF-κB phosphorylations and activated JAK2/STAT3 pathway [11, 33, 34]. We showed here that compared with H/R, H/R + NaHS decreased ROS levels and apoptosis, down-regulated NF-κB and JAK2-STAT3 pathways. PC + NaHS further enhanced the effects of H/R + NaHS. The effect of NaHS was similar to NAC (an inhibitor of ROS), PDTC (a NF-κB inhibitor), AG 490 (a JNK2 inhibitor) during PC, respectively (Figs. 2, 3, 4, 5, 6, 7, 8). Taken together, these findings suggest that exogenous H_2_S plays an important role in the recovery of PC-induced cardioprotection by inhibiting ROS generation through decrease of NF-κB and JAK2-STAT3 pathways.

## Conclusion

**Fig. 9:**
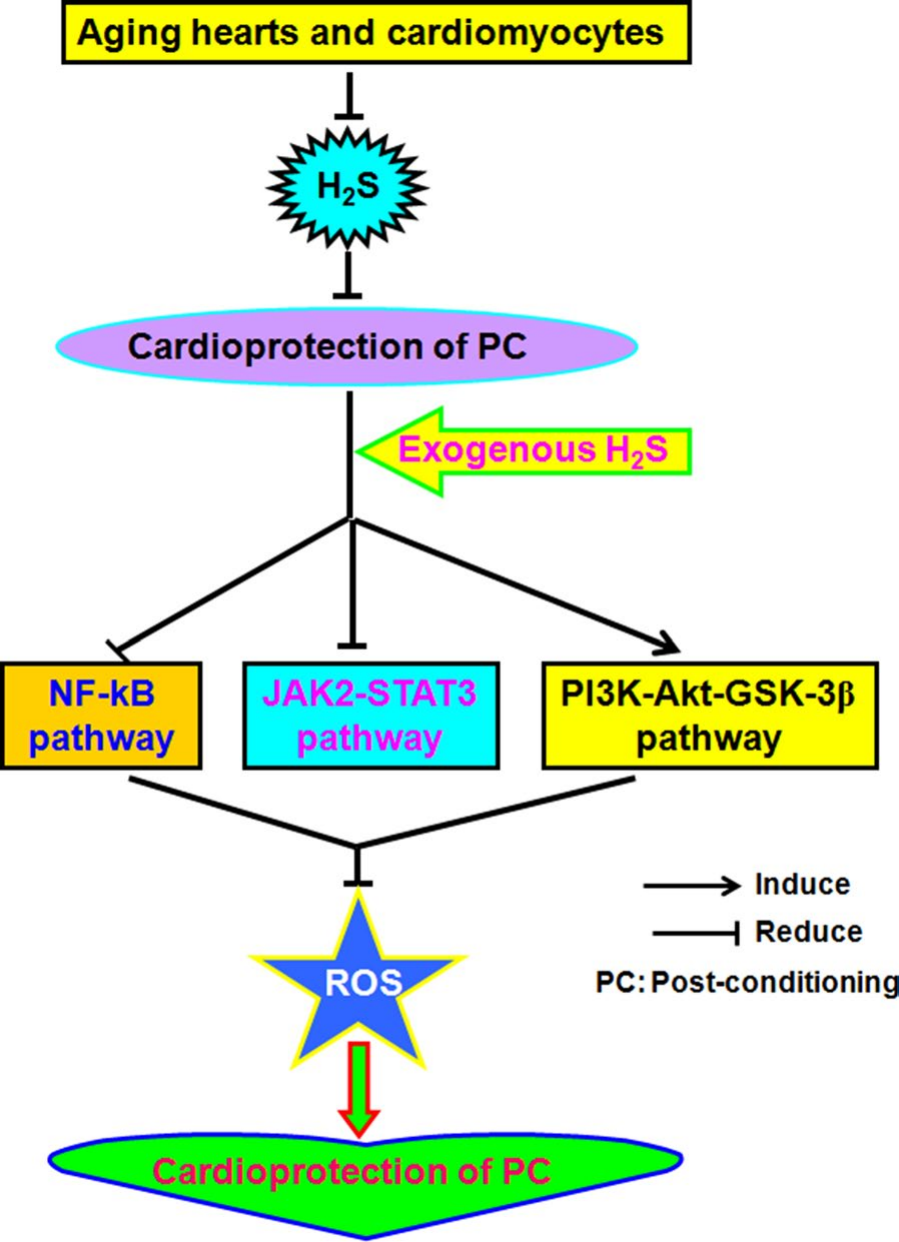
Involvement of exogenous H_2_S in recovery of PC-induced cardioprotection in the aging cardiomyocytes. Exogenous H_2_S mediates recovery of PC-induced cardioprotection via decrease of ROS level by down-regulation of NF-κB and JAK2-STAT3 pathways and up-regulation of PI3K-Akt-GSK-3β pathway in the aging hearts and cardiomyocytes

In conclusion, previous study and current results indicated that exogenous H_2_S contributes to recovery of ischemic post-conditioning-induced cardioprotection by decrease of ROS levels via down-regulation of NF-κB and JAK2-STAT3 pathways and up-regulation of PI3K-Akt-GSK-3β pathway in the aging hearts and cardiomyocytes (Fig. 9).
